# Hematocrit, age, and survival in a wild vertebrate population

**DOI:** 10.1002/ece3.7015

**Published:** 2020-12-21

**Authors:** Thomas J. Brown, Martijn Hammers, Martin Taylor, Hannah L. Dugdale, Jan Komdeur, David S. Richardson

**Affiliations:** ^1^ School of Biological Sciences University of East Anglia Norwich UK; ^2^ Groningen Institute for Evolutionary Life Sciences University of Groningen Groningen The Netherlands; ^3^ School of Biology Faculty of Biological Sciences University of Leeds Leeds UK; ^4^ Nature Seychelles Victoria Mahé Seychelles

**Keywords:** aging, biomarkers, birds, condition markers, hematocrit, life history, senescence, survival, trade‐offs, wild populations

## Abstract

Understanding trade‐offs in wild populations is difficult, but important if we are to understand the evolution of life histories and the impact of ecological variables upon them. Markers that reflect physiological state and predict future survival would be of considerable benefit to unraveling such trade‐offs and could provide insight into individual variation in senescence. However, currently used markers often yield inconsistent results. One underutilized measure is hematocrit, the proportion of blood comprising erythrocytes, which relates to the blood's oxygen‐carrying capacity and viscosity, and to individual endurance. Hematocrit has been shown to decline with age in cross‐sectional studies (which may be confounded by selective appearance/disappearance). However, few studies have tested whether hematocrit declines within individuals or whether low hematocrit impacts survival in wild taxa. Using longitudinal data from the Seychelles warbler (*Acrocephalus sechellensis*), we demonstrated that hematocrit increases with age in young individuals (<1.5 years) but decreases with age in older individuals (1.5–13 years). In breeders, hematocrit was higher in males than females and varied relative to breeding stage. High hematocrit was associated with lower survival in young individuals, but not older individuals. Thus, while we did not find support for hematocrit as a marker of senescence, high hematocrit is indicative of poor condition in younger individuals. Possible explanations are that these individuals were experiencing dehydration and/or high endurance demands prior to capture, which warrants further investigation. Our study demonstrates that hematocrit can be an informative metric for life‐history studies investigating trade‐offs between survival, longevity, and reproduction.

## Graphical abstract

Using long‐term data we investigated the association between haematocrit, longitudinal age and survival in a wild vertebrate population—the Seychelles warbler (*Acrocephalus sechellensis*). Haematocrit exhibited a distinctive age‐dependent pattern within‐individuals, and young individuals with high haematocrit had poorer survival prospects. This study highlights a novel physiological change with age that is indicative of individual condition in wild populations.

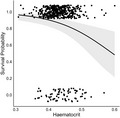

## 1 INTRODUCTION

An organism's fitness is the product of many integrated physiological systems, and their interaction with the environment. Activity in one physiological system can limit resource availability and generate negative consequences (e.g., by‐products) for another (Harshman & Zera, 2007; Leroi, 2001; Ricklefs & Wikelski, 2002). These trade‐offs form the basis of life history. Physiological markers provide valuable insights into life‐history trade‐offs, condition, and senescence—particularly in wild populations, where complex environmental factors can weaken associations between life‐history traits and observed fitness (Nussey et al., 2008). Many physiological markers (oxidative stress, hormone regulation, etc.) yield complex and/or inconsistent associations with life history, survival, and reproductive success (e.g., Johnstone et al., 2017; Norris & Evans, 2000; Speakman & Selman, 2011; Wilder et al., 2016); thus, there is a continued need to identity and validate such markers.

Aerobic capacity, which contributes to endurance and performance, is a vital physiological trait for organismal health and fitness. Aerobic capacity depends on the oxygen‐carrying capacity of blood, which is determined by the concentration of hemoglobin and the rate of blood flow, which is inversely proportional to blood viscosity (Birchard, 1997; Calbet et al., 2006; Wagner, 1996). These properties are reflected by hematocrit or packed cell volume (PCV); the proportion of whole‐blood volume is comprised of erythrocytes. Hemoglobin and blood viscosity increase linearly and exponentially, respectively, with hematocrit (Hedrick et al., 1986). Blood becomes harder to circulate with increasing viscosity (i.e., requiring greater cardiovascular effort), but less viscous blood contains less hemoglobin. Therefore, intermediate hematocrit levels (*ca*. 40%) are optimal for maximum oxygen‐carrying capacity and endurance (Birchard, 1997; Jensen et al., 2013; Schuler et al., 2010).

Hematocrit levels observed in nature are variable (range *ca*. 30%–60%) within and between endothermic species (Stark & Schuster, 2012). For example, hematocrit is higher in species/individuals requiring greater blood oxygen storage and endurance (Lourdais et al., 2014; Minias, 2015; Yap et al., 2019). Similarly, hematocrit tends to increase within individuals in response to elevated oxygen demands, such as during altitudinal migration (Borras et al., 2010) and exercise regimes (reviewed in Yap et al., 2017). Elevated hematocrit occurs via the production of new erythrocytes (erythropoiesis) and/or the release of reticulocytes (immature erythrocytes) from the bone marrow, which is triggered by hypothalamus–pituitary–adrenal‐mediated stress (see Voorhees et al., 2013). More rapid (<1 hr) increases in hematocrit can occur due to a reduction in blood plasma volume (hemoconcentration), which happens during exercise and dehydration (Bury et al., 2019; Kaltreider & Meneely, 1940). In some mammal species, splenic reservoirs of erythrocytes can also increase hematocrit at the onset of stress and exercise (Böning et al., 2011).

Anemia—characterized by chronically low hematocrit and hemoglobin—occurs when an individual's rate of erythrocyte loss exceeds that of erythropoiesis, for example, during blood parasitism (O’Brien et al., 2001). However, anemia can occur without affecting hematocrit, since the release of reticulocytes, which are larger than mature erythrocytes, can rapidly complement hematocrit despite them having lower hemoglobin content (Fair et al., 2007). Anemia can also arise as a secondary outcome of competing physiological systems. For example, egg production in birds causes a reduction in hematocrit via an estrogen‐mediated suppression of erythropoiesis and hemodilution—an increase in blood plasma volume (Wagner, Prevolsek, et al., 2008; Wagner, Stables, et al., 2008; Williams et al., 2004). Therefore, both within‐individual increases and decreases in oxygen‐carrying capacity and associated factors (hematocrit and hemoglobin) have the potential to reflect a multitude of life‐history events and trade‐offs (for reviews, see Fair et al., 2007; Johnstone et al., 2017; Minias, 2015).

Uncertainty remains regarding associations between hematocrit, age, and senescence in wild animals. From birth to maturity, hematocrit increases with age (e.g., Cornell & Williams, 2017; Eklom & Lill, 2006; Trillmich et al., 2008), but few studies have determined the age dependence of hematocrit in adult life. This likely stems from the difficulty of obtaining samples of known‐age adults in many wild systems. In captive mice and humans, low hematocrit in extreme old age reflects senescence in erythrocyte renewal mechanisms (Boggs & Patrene, 1985; Gaskell et al., 2008). Similarly, cross‐sectional studies of other captive and wild vertebrates have observed lower hematocrit in old age, suggestive of senescence (Elliott et al., 2015; Jégo et al., 2014; Prinzinger & Misovic, 2010; Smucny et al., 2004). However, such observations may also arise from compositional changes in successive age classes of a population, for example, due to selective disappearance of individuals with high hematocrit. Longitudinal studies are needed to explicitly investigate within‐individual change with age (Elliott et al., 2015; Nussey et al., 2008).

Factors that cause hematocrit to deviate from the theoretical optimum (for oxygen‐carrying capacity and general health) could have long‐term impacts on the fitness of wild taxa. For example, experimental reductions in hematocrit in birds can result in reduced reproductive success (Fronstin et al., 2016) and flight performance (Yap et al., 2018). However, few studies have investigated associations between hematocrit levels observed under natural conditions in the wild and subsequent survival. Anemia results in lethargy and fatigue, but even minor decreases in oxygen‐carrying capacity could represent an energetic disadvantage that reduces survival prospects in wild settings. Conversely, more viscous blood, and the cardiovascular loading this creates, is linked to negative health impacts in humans (Brækkan et al., 2010; Coglianese et al., 2012; Stack & Berger, 2009; Walton et al., 2017). Extreme high or low hematocrit can also be a noncausal indicator of factors detrimental to self‐maintenance, such as stress, parasitism, and nutrient deficiencies (see Johnstone et al., 2017). Therefore, intermediate hematocrit levels are expected to be optimal for survival (e.g., Boffetta et al., 2013; Bowers et al., 2014).

The isolated Seychelles warbler (*Acrocephalus sechellensis*) population on Cousin Island provides an excellent model system for studying associations between hematocrit, age, and survival in a wild population. This system benefits from over 30 years of continuous monitoring and extremely accurate survival estimates of known‐age individuals that are not confounded by dispersal (Hammers et al., 2019; Komdeur, 1992; Richardson et al., 2007). Individuals have been captured and blood sampled repeatedly across their lifetime, providing a wealth of longitudinal physiological data (Hammers et al., 2015), including hematocrit. Here, we first assess the relationship between hematocrit and age. Based on previous findings across vertebrate taxa, we predict that hematocrit increases during early life up to maturity, followed by an age‐related decline. Crucially, we determine the relative contribution of longitudinal (i.e., within‐individual) and cross‐sectional (i.e., between‐individual) effects to any age patterns observed. Hematocrit is also likely to vary between and within individuals independently of age. We determine whether this variation is explained by other factors, namely sex and social status and breeding stage, and assess within‐individual repeatability of hematocrit. Lastly, we determine the relationship between hematocrit and annual survival probability. Given the potentially negative effects of both low and high hematocrit, we predicted that individuals with intermediate hematocrit values would have higher survival. Our study will therefore assess the validity of hematocrit as a marker of condition within wild animal populations, and explore its usefulness in terms of providing insights into the costs and trade‐offs that individual animals face during life.

## 2 METHODS

### 2.1 Study species and data collection

The Seychelles warbler is a small insectivorous passerine endemic to the Seychelles. Seychelles warblers can (exceptionally) reach ages of up to 19 years (Hammers & Brouwer, 2017), though the average life span is 5.5 years for individuals that reach fledgling age (Komdeur, 1991). The population of *ca*. 320 adult individuals on Cousin Island (29 ha, 4°209 S, 55°409 E) has been extensively monitored since 1986. Monitoring is carried out for *ca*. 6 months of each year (January–March, June–September) during the minor and major breeding seasons, respectively (Komdeur & Daan, 2005). Since 1997, nearly all individuals (>96%) have been ringed with a unique combination of a British Trust for Ornithology (BTO) metal ring and three color rings for identification (Richardson et al., 2001). Individuals are usually first caught and ringed as nestlings or dependent fledglings, before sexual maturity (<8 months old). Juveniles are assigned to age categories (fledgling 1–3 months, old fledgling 3–5 months, or subadult 5–8 months), based on behavior and eye color, which transitions from gray in fledglings to red‐brown in adults (Komdeur, 1992).

The population is structured into clearly defined territories that are defended year round. Breeding groups is comprised of one socially monogamous dominant pair (hereafter dominant breeders), but may also include 1–5 sexually mature subordinates (Richardson et al., 2002) which sometimes engage in helping behavior and cobreeding (Hammers et al., 2019). An Individual's social status in a given field season is determined through observations of behavior (see Komdeur, 2001).

During the breeding season, each territory is visited at least every 2 weeks and checked for the presence–absence of individuals identified by their color ring combination. Dominant females are followed for 15 min to determine whether an active nest is present. Once a nest is found, it is visited every 3 days for 15–60 min (to determine breeding stage) until completion or failure. For nests that were discovered during or after the start of incubation, the egg‐laying date is estimated from the timing of hatching (determined from provisioning observations) and/or fledging. Given that interisland dispersal is exceptionally rare (Komdeur et al., 2004) and resighting probabilities are close to one (Brouwer et al., 2009), birds that are not seen during a field season can be assumed dead (Hammers et al., 2013). The last day of a field season for which an individual is observed as present is taken as the date of death.

Individuals were captured using mist nets and conspecific playback (see Kingma et al., 2016 for details). *Ca*. 70 μl of blood was drawn with a microcapillary tube from the brachial vein. A small amount (*ca*. 10 ul) of blood sample was also stored in absolute ethanol at 4°C for future DNA extraction. This procedure is the routine, nonlethal way to sample blood from passerine birds and has been shown to have no measurable effect on condition or survival (Sheldon et al., 2008). Within *ca*. 3 hr of bleeding, microcapillary tubes were centrifuged for 8 min at 6,000 *g* to separate erythrocytes from plasma, white blood cells, and platelets. Hematocrit was measured (using sliding calipers ±0.01 mm) as the proportion of erythrocytes relative to whole‐blood volume. Between the years of 2003 and 2017, 1,383 hematocrit measurements were obtained from 733 individuals. DNA was extracted using a salt extraction technique following Richardson et al. (2001), and sex of the individual was confirmed using the PCR‐based method outlined by Griffiths et al. (1998).

### 2.2 Statistical analyses

All statistical analyses were performed with RStudio (v1.2.5033, RStudio Team, 2020). Firstly, we investigated the relationship between hematocrit and age across all samples with a generalized additive mixed model (GAMM) using the gamm4 package (v0.2–6). In this model, we fitted a nonparametric smoothing parameter for age to evaluate expected nonlinear relationships between hematocrit and age. Compared with linear mixed models (LMMs), which require prespecified functions between dependent and continuous predictor variables, GAMMs are more appropriate when the shape of age‐dependent patterns are unknown (Hammers et al., 2016). In addition to age, the model included factors known to influence hematocrit in avian taxa (see Fair et al., 2007): sex, social status (dominant breeders vs. subordinates + juveniles), and time of day of sampling. Sex differences are likely to depend on social status; thus, a two‐way interaction between sex and status was included. To control for nonindependent samples, individual identity, breed group identity, and catch year were included as random intercepts.

Age‐related patterns across populations can arise from selective disappearance, whereby certain phenotypes are associated with shorter life spans (Nussey et al., 2008). To control for selective disappearance effects, we repeated the model using only individuals that were dead at the time of analysis and included age at death as an additional factor (de Pol & Verhulst, 2006; van Hammers et al., 2019).

Our GAMM analysis revealed that dominant females had significantly lower hematocrit than dominant males and subordinates (male or female). This suggested an effect of reproductive anemia on hematocrit levels of dominant females, since they produce the majority of offspring and sampling coincided with the breeding seasons. To determine whether sex‐by‐status differences were maintained in individuals not engaged in reproduction, we repeated the model including only sexually mature individuals (>8 months old) sampled outside of known breeding attempts; either no egg was laid for that breed group or the individual was sampled >50 days from the breed groups lay date. For a given individuals breed group, we calculated the number of days between the estimated lay date and the date of sampling. For breed groups with two or more broods (which occurs if, e.g., the first brood was predated), the closest lay date from the sample date was selected. For individuals sampled during breeding attempts (<50 days from breed groups lay date), we expected hematocrit to be lowest nearer the lay date, and only in dominant females. Since hematocrit was expected to fluctuate nonlinearly across breeding stages, nonparametric smoothing parameters were fitted for days from lay date for males and females. Separate models were created for dominant breeders and subordinates to avoid the need for complex three‐way interactions between sex, status, and days from lay date. Individuals were rarely caught multiple times within the same breeding attempt; thus, this section of our analysis is cross‐sectional in nature.

**Figure 1:**
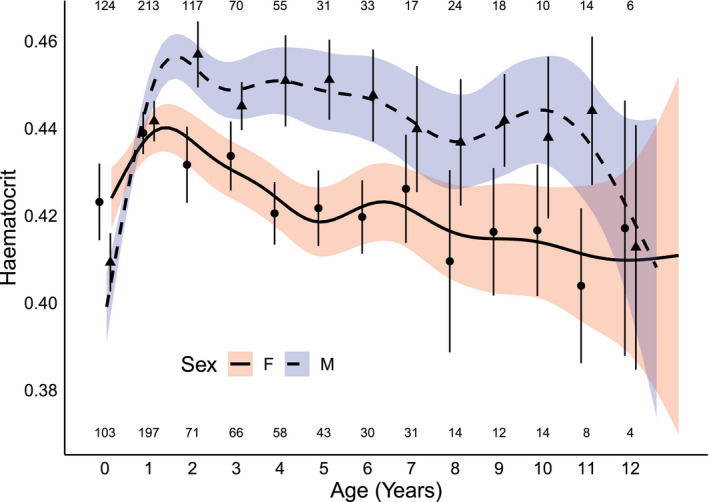
Hematocrit in Seychelles warblers in relation to age and sex. The fit lines (solid = female, dashed = male) show nonparametric smoothing functions for age with 95% confidence intervals. Points (round = female, triangles = male) are means and 95% confidence intervals for each age (rounded years). Ages 12 and 13 are grouped for graphical purposes (denoted as 12 here). Within‐graph numbers represent sample sizes per age for females (lower) and males (upper)

To separate the role of between‐ versus within‐individual variation with age (i.e., cross‐sectional from longitudinal effects), we used the within‐subject centering method described by van de Pol & Wright, 2009. Briefly, age at sampling is split into two predictors, (a) mean age across all sampling events for a given individual (mean age), and (b) within‐individual deviation from mean age (∆ age). Our GAMM indicated a peak in hematocrit at *ca*. 1.5 years of age (see Results, Figure 1). To investigate the initial increase and subsequent decrease in hematocrit in more detail, we performed within‐individual centering for individuals <1.5 months and for individuals ≥1.5 months in separate analyses (following Hammers et al., 2016). This allowed us to compare the drivers of age‐related hematocrit patterns in early life versus later adulthood.

**Figure 2:**
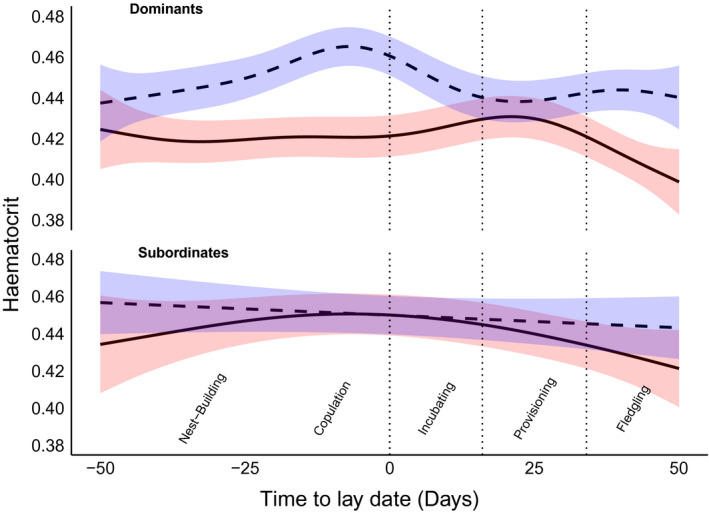
Hematocrit in Seychelles warblers in relation to days from lay date for dominant breeders (left) and subordinates (right). The fit lines (solid = female, dashed = male) show non‐parametric smoothing functions for days from lay date, and the shaded area is the 95% confidence interval for the smoothing functions. The annotations and dotted lines denote theoretical nest stages relative to lay date

We created linear mixed models using the lme4 package (v1.1‐21, Bates et al., 2014) with hematocrit as the response and mean age, ∆ age, sex, social status, and time of day of sampling as predictors. Age terms were entered as both linear and quadratic terms to test for possible nonlinear patterns. Two‐way interactions between ∆ age, sex, and status were included to determine whether within‐individual changes in hematocrit were dependent on these factors. Consistent with the GAMMs outlined above, individual identity, breed group identity, and catch year were included as random intercepts. Due to the relationship observed between hematocrit and breeding stage in dominant breeders (see Figure 2), we repeated the analysis excluding samples from breeding stages where hematocrit deviates from typical levels; 20 days before to 5 days after laying for dominant males, and 30–50 days after laying in dominant females. Using the rptR package (v0.9.22; Nakagawa & Schielzeth, 2010), we also calculated repeatability estimates for hematocrit within individuals to determine how consistent individual hematocrit levels are across repeated samples at different times.

Lastly, we investigated whether hematocrit predicts short‐term survival. We used a binominal generalized linear mixed models (GLMMs) to test the probability of surviving 1 year beyond the date of sampling (Y/N) in relation to hematocrit. Since hematocrit exhibited different age‐specific patterns in early life (<1.5 years) and adulthood (1.5–13 years; see Results), we investigated the relationship between hematocrit and survival for these two age groups in separate models. Where multiple hematocrit samples were taken per individual, only the last sample was selected, which allowed us to identify whether individuals facing imminent mortality have different hematocrit levels compared with those that survive. Additional fixed effects included age, sex, status, and quadratic functions of hematocrit and age. We also included an interaction term between age and hematocrit to see whether the effect of hematocrit on survival changed with age. Survival probability can vary between territories and years, for example, due to varying food availability (Brouwer et al., 2006; Spurgin et al., 2017); thus, breed group and catch year were also entered as random factors. Breed group was subsequently dropped as a random factor in the 1.5‐ to 13‐year group due to model convergence issues. As with the LMM analysis, we repeated models excluding samples from breeding stages where hematocrit deviates from typical levels in dominant breeders.

In all models, nonsignificant interaction terms were removed sequentially (in order of least significance) and only reported if of specific interest. All fixed effects remained in final models (regardless of significance) except for quadratic functions of continuous variables, which were removed when nonsignificant (see Whittingham et al., 2006). Parameter estimates and significance of removed effects were determined by re‐entering them into final models.

## 3 RESULTS

### 3.1 Cross‐sectional age

Hematocrit had a distinctive pattern with cross‐sectional age (Figure 1). Initially, hematocrit increased rapidly before reaching a peak at *ca*. 1.5 years of age. From 1.5 years onward (maximum age in this analysis is 13 years), hematocrit showed a consistent downward trajectory (Figure 1). This age‐dependent pattern was similar for both sexes (Figure 1) and fitting smoothed age terms for males and females separately resulted in poorer model fit (∆AIC > 4). Sex differences in hematocrit were dependent on social status (Table 1). For dominant breeders, which are the vast majority of individuals sampled ≥2 years of age, females had lower average hematocrit levels than males. Dominant females also had lower hematocrit than subordinates (male or female; Table 1, Figure S1). Individuals sampled in the early morning had higher hematocrit than those sampled in the late afternoon (Table 1). When the model was run on a subset of individuals known to be dead, including age at death as a predictor, we found that shorter‐lived individuals had significantly higher hematocrit (Table S1). Therefore, selective disappearance of individuals with high hematocrit contributed to the age‐specific pattern. Crucially, the effect of age was still significant when controlling for age at death (Table S1), indicating that within‐individual effects were also present.

**Table 1:** Hematocrit in relation to cross‐sectional age and other factors in Seychelles warblers

Predictor	*β*	*SE*	*t*	*p*
Intercept	0.460	0.005	94.701	<.001
Sex (male)	−0.003	0.003	−1.202	.230
Status (dominant)	**−0.021**	**0.003**	**−6.440**	**<.001**
Sample time	**−0.002**	**0.000**	**−5.981**	**<.001**
Sex × Status	**0.028**	**0.004**	**7.612**	**<.001**

Smoothed terms	*df*	*F*	*p*
Age	**7.805**	**16.73**	**<.001**

Random factors	1,379 observations	Variance
Individual identity	730 individuals	<0.000
Breed group	747 breed groups	<0.000
Catch year	14 years	<0.000

Note

Results are from a GAMM analysis with a nonparametric smoothing parameter for age. Significant effects are in bold.

### 3.2 Reproductive stage

We compared hematocrit of sexually mature (>8 months old) subordinates and dominant breeders. Outside of breeding attempts, dominant females had lower hematocrit than both dominant males and subordinate males and females (Table S2, Figure S1). During breeding attempts, the effects were more complex (Table S3). There was no evidence of reproductive anemia (i.e., a marked decrease in hematocrit) in dominate females sampled near their lay date, although hematocrit was lower at *ca*. 35–50 days after laying (Figure 2). The hematocrit of males exhibited a complex relationship with breed group lay date; hematocrit was highest at *ca*. 7 days prior to laying and was lowest 15–30 days postlaying (Figure 2). In contrast to dominant breeders, there was no significant difference between male and female subordinates sampled during breeding attempts (Tables S3 and S4, Figure 2). The hematocrit of subordinate males did not vary in relation to breed group lay date, but subordinate females exhibited a weak quadratic relationship with days from breed group lay date; peaking at the laying date (Figure 2). Importantly, the decline of hematocrit with increasing age persisted when the analysis was split between nonbreeding and breeding individuals, and (for the latter) when controlling for days from lay date (Figure S2).

### 3.3 Longitudinal age

**Figure 3:**
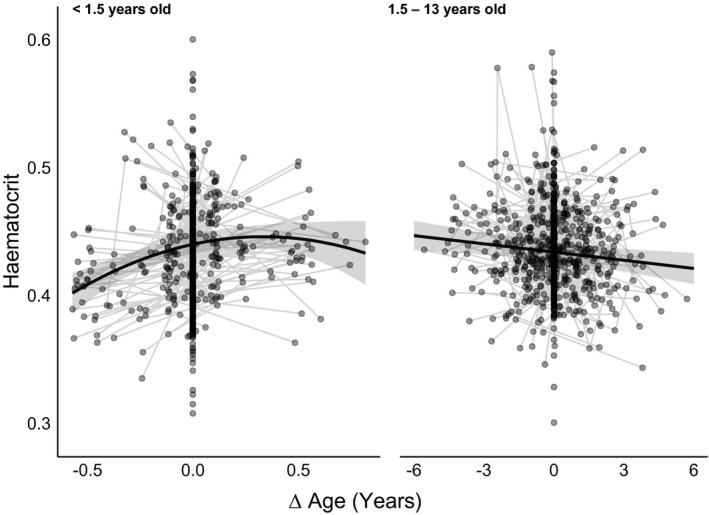
Hematocrit in Seychelles warblers in relation to within‐individual differences in age (∆ Age) for individuals <1.5 years old and 1.5–13 years old. Thick black lines are the LMM (Table 2) predicted hematocrit ±95% CI relative to age. Raw data are hematocrit samples with thin gray lines connecting multiple samples from the same individual

Our within‐subject centering analysis, which separates within‐ and between‐individual contributions to age patterns, was consistent with the GAMM analysis. Below 1.5 years of age, hematocrit increased both within and between individuals with age in a quadratic pattern; a strong initial increase that plateaued at *ca*. 1 year of age (Table 2, Figure 3). From 1.5 years of age onward, hematocrit declined linearly with increasing age both within and between individuals (Table 2, Figure 3). All interactions with ∆ age were nonsignificant; thus, within‐individual increases (<1.5 years) and decreases (1.5—13 years) did not vary between individuals of differing sex or status. Consistent with the GAMM analysis, hematocrit was lower in dominant females and individuals caught later in the day (Table 2). All results were qualitatively identical when samples from dominant breeders caught during key breeding stages (where hematocrit deviated from typical levels; see Figure 2) were excluded from the analysis (Table S5).

**Table 2:** Hematocrit in relation to cross‐sectional age (mean age) and longitudinal age (∆ age) in Seychelles warbler <1.5 years old and 1.5–13 years old

Predictor	*β*	*SE*	*t*	*p*
<1.5 years old				
Intercept	0.393	0.010	40.92	<.001
Mean age	**0.159**	**0.022**	**7.327**	**<.001**
Mean age^2^	**−0.085**	**0.016**	**−5.436**	**<.001**
∆ Age	**0.036**	**0.008**	**4.437**	**<.001**
∆ Age^2^	**−0.054**	**0.018**	**−3.09**	**.002**
Sex (male)	−0.005	0.003	−1.615	.107
Status (dominant)	**−0.014**	**0.007**	**−2.061**	**.040**
Sample time	**−0.002**	**0.000**	**−3.276**	**.001**
Sex × Status	**0.017**	**0.008**	**2.143**	**.033**

Random factors	637 observations	Variance
Individual identity	506 individuals	<0.000
Breed group	452 breed groups	<0.000
Catch year	14 years	<0.000

Predictor	*β*	*SE*	*t*	*p*
1.5–13 years old				
Intercept	0.465	0.006	74.692	<0.001
Mean age	**−0.002**	**0.001**	**−3.608**	**.000**
∆ Age	**−0.002**	**0.001**	**−2.715**	**.007**
Sex (male)	**0.026**	**0.002**	**10.677**	**<.001**
Status (dominant)	**−0.013**	**0.003**	**−3.619**	**<.001**
Sample time	**−0.002**	**0.000**	**−4.714**	**<.001**

Random factors	742 observations	Variance
Individual identity	405 individuals	<0.000
Breed group	529 breed groups	<0.000
Catch year	14 years	<0.000

Note

Parameters shown are from LMM analysis. Significant effects are in bold.

For final LMMs (excluding all nonsignificant interactions and nonsignificant quadratic effects), we calculated repeatability of hematocrit within individuals. For individuals below 1.5 years of age, only 1.6% (*p* = .434) of variance was due to within‐individual consistency. From 1.5 years of age, within‐individual consistency was higher at 7.8% and approaching significance (*p* = .063).

### 3.4 Survival

**Figure 4:**
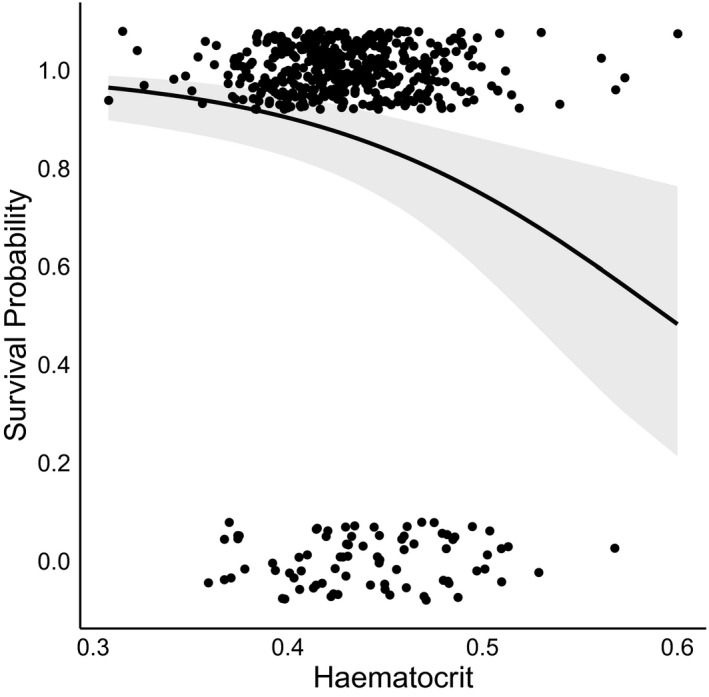
The probability of young (<1.5 years of age) Seychelles warblers surviving one year after sampling relative to haematocrit. The fit‐line is a binomial regression between survival (Y/N) and haematocrit with 95% confidence limits. Raw data depicts the distribution of observed survival counts (1 = survived, 0 = deceased)

A total of 263 out of the 1,383 samples taken were from individuals that died within the subsequent year. For young (<1.5 years of age), individuals with higher hematocrit were less likely to survive to the next year (Table 3, Figure 4). In contrast, hematocrit did not predict survival over the subsequent year for individuals 1.5–13 years of age (Table 3). Contrary to expectations, there was no quadratic effect of hematocrit on survival; only high hematocrit was associated with lower survival in young individuals. The effect of hematocrit on survival was not influenced by age in either age category. Survival probability was lower for males and subordinates from 1.5–13 years of age (Table 3). Repeating the analysis while excluding samples from dominant breeders caught at key breeding stages (where hematocrit deviated from typical levels; Figure 2) did not qualitatively change results (Table S6).

**Table 3:** Survival in the Seychelles warbler in relation to haematocrit for individuals 1.5 years old and 1.5–13 years old

Predictor	*β*	*SE*	*z*	*p*
<1.5 years old				
Intercept	5.016	1.283	3.909	<.001
Hematocrit	**−9.293**	**3.004**	**−3.093**	**.002**
Sex (male)	−0.224	0.224	−1.001	.317
Status (dominant)	0.565	0.369	1.533	.125
Age	0.459	0.395	1.161	.246

Random factors	506 observations	Variance
Breed group	418 breed groups	<0.000
Catch year	14 years	0.340

Predictor	*β*	*SE*	*z*	*p*
1.5–13 years old				
Intercept	1.485	1.668	0.89	.3733
Hematocrit	−1.398	3.684	−0.379	.7044
Sex (male)	**−0.557**	**0.254**	**−2.196**	**.0281**
Status (dominant)	**0.724**	**0.345**	**2.099**	**.0358**
Age	−0.009	0.043	−0.202	.8403

Random factors	408 observations	Variance
Catch year	13 years	0.370

Note

Results are from binominal GLMMs with survival to the following year (Y/N) as the response variable. Significant effects are in bold.

## 4 DISCUSSION

Hematocrit increased in juveniles up to 1.5 years of age, beyond which hematocrit declined with increasing age. Both longitudinal change and the selective disappearance of individuals with high hematocrit contributed to this age‐specific pattern. In early life (<1.5 years), hematocrit increased within individuals, but individuals with higher hematocrit were less likely to survive to the following year. After 1.5 years of age, hematocrit declined within individuals with advancing age. However, hematocrit did not predict survival in this older age range. In addition to age, hematocrit was lower in females compared with males, but only in dominant breeders (hematocrit did not significantly differ between subordinates and dominant males). The hematocrit of dominant breeders also varied with breeding stage—most notably, male hematocrit peaked in the days prior to the pair‐bonded females lay date. However, the relationship between hematocrit with both age and survival persisted when samples from dominant individuals caught during key breeding stages were excluded from analyses.

### 4.1 Age

Increases in hematocrit and oxygen‐carrying capacity during development have been observed in a range of vertebrates (Fair et al., 2007; Petschow et al., 1978; Trillmich et al., 2008). In birds, adult levels of hematocrit are usually achieved at late‐nestling age, presumably in preparation for fledging (Eklom & Lill, 2006a, 2006b). However, in the Seychelles warbler hematocrit continues to increase postfledging (up to 1.5 years). Our longitudinal and survival analyses confirmed that increases in hematocrit occurred within juveniles and were not due to juveniles with low hematocrit having lower annual survival, that is, selective disappearance. In this species, fledglings receive parental care (provisioning) for up to 3 months (Komdeur, 1996) and may delay dispersal from the natal territory (i.e., become subordinates) for 1–3 years (Hammers et al., 2013). Socially dominant individuals are expected to have higher aerobic demands (for territory defense and reproduction) than juveniles, which might explain why hematocrit increases up to 1.5 years of age. Interestingly, hematocrit levels at this age (1–3 years) were higher than levels across prime reproductive ages (*ca*. 4–7 years; Komdeur, 1996; Hammers et al., 2012), which could reflect elevated oxygen demands and/or stress in subordinates competing for dominant social positions at this age (Creel, 2001; Kingma et al., 2016).

We observed a gradual decrease in hematocrit within individuals with advancing age during most of adulthood. This relationship is similar to that observed in thick‐billed murres (*Uria lomvia*) by Elliott et al. (2015), the only other longitudinal study of hematocrit in a wild system to date, and concurs with results from cross‐sectional studies. For example, Jégo et al. (2014) found a decrease in hematocrit in roe deer (*Capreolus capreolus*) from the onset of actuarial senescence (8 years old). Thus, declining hematocrit could be indicative of physiological senescence (i.e., diminished ability to maintain circulating erythrocyte mass) in adult Seychelles warblers. By restricting endurance, such declines could contribute to lower survival in elderly life; onset of actuarial senescence in the Seychelles warbler is *ca*. 7 years old (Hammers et al., 2015). However, we did not find evidence of this in our study since only high hematocrit (not low hematocrit) was associated with lower survival, and only in young individuals. Alternatively, decreases in hematocrit could reflect other behavioral and/or physiological changes with age observed in wild vertebrates. For instance, gains in experience could relax demands for oxygen‐carrying capacity during foraging (Daunt et al., 2007; Zimmer et al., 2011). Furthermore, the intensity of stress responses—which can elevate hematocrit (Johnstone et al., 2012)—often decline with age (Lendvai et al., 2015; Wilcoxen et al., 2011). Such changes may be expected of older Seychelles warblers living in long‐established territories.

### 4.2 Sex and reproduction

We found that hematocrit was lower in female, compared with male, Seychelles warblers, but only for dominant breeders. This suggests an effect of reproduction on hematocrit, given that dominant breeders produce the vast majority of offspring in the population (Raj Pant et al., 2019; Richardson et al., 2001) and sampling coincided with peaks in breeding activity (Komdeur & Daan, 2005). A well‐documented phenomenon (see Fair et al., 2007) in female birds is reproductive anemia—a reduction in hematocrit during egg laying due to the pleiotropic effects of elevated estrogen (Wagner, Prevolsek, et al., 2008; Wagner, Stables, et al., 2008; Williams et al., 2004). Hematocrit declines observed in other species range from 5% to 10% (Davey et al., 2000; Morton, 1994) and can persist for several weeks, through incubation and chick‐rearing (Williams et al., 2004). In contrast to females, males can have elevated hematocrit prior to and during reproduction as a consequence of elevated testosterone, which stimulates erythropoiesis (Mirand et al., 1965). Therefore, sex differences may only occur during reproduction (e.g., Morton, 1994). This was not the case in our study since sex‐by‐status differences were similar both during and outside of breeding attempts. Additionally, there was no evidence of reproductive anemia (low hematocrit at egg laying) in dominant females. Taken together, these findings indicate that dominant females maintain hematocrit levels at a constant low level (relative to dominant males and subordinates of either sex). In other species, estrogen is positively related to territorial behaviors (e.g., singing and aggression) in females (Pärn et al., 2008; Woodley & Moore, 1999). This suggests that female dominance in Seychelles warblers might be accompanied by an upregulation of estrogen, which subsequently lowers hematocrit. However, we do not currently have data on estrogen dynamics in this species.

Sex differences in hematocrit were greatest prior to egg laying due to increased hematocrit in dominant males. Peak dominant male hematocrit coincided with his female partners fertile period (6 days prior to egg laying), during which testosterone levels of (pair‐bonded) dominant males is also highest (Van De Crommenacker et al., 2004). Therefore, elevated hematocrit in males may be a consequence of elevated testosterone (e.g., Buttemer & Astheimer, 2000; Ezenwa et al., 2012). A lack of elevated hematocrit in subordinate males supports this explanation, since subordinate males do not elevate testosterone levels during the female fertile period (Van De Crommenacker et al., 2004). Elevated hematocrit might also reflect broader behavioral and physiological changes during this period. For example, dominant—but not subordinate—males invest in energetically costly guarding of mates during their fertile period to prevent extra‐pair copulations (Komdeur, 2001). Thus, elevated hematocrit could reflect increased activity levels—and therefore higher oxygen demands—during this critical period for dominant males (see Hammond et al., 2000).

### 4.3 Survival

Intermediate hematocrit levels are predicted to be advantageous for survival, given that both high and low hematocrit are associated with increased mortality in humans and mice (Boffetta et al., 2013; Heller et al., 1998; Wagner et al., 2001). However, we found young Seychelles warblers with low hematocrit had the highest survival. This finding contradicts a study by Bowers et al. (2014), which found that house wren (*Troglodytes aedon*) nestlings with intermediate hematocrit had higher recruitment. However, in this study a more extreme lower range of hematocrit values (i.e., <30%) was apparent, likely due to age; neonates having lower hematocrit compared with juveniles and adults. Extreme‐low hematocrit in neonates likely reflects developmental immaturity, which would reduce the probability of successful fledging (Cornell et al., 2017).

In adulthood, low hematocrit can be indicative of anemia (Campbell, 1994), which in wild populations may increase mortality risk via lethargy and fatigue. However, these symptoms are also likely to preclude anemic individuals from being captured using mist nets. In our sample, only 11 individuals were caught with what are considered anemic hematocrit levels in captive avifauna (<35%; Campbell, 1994). However, the threshold of anemic hematocrit might be higher in wild populations, given that overall hematocrit can be higher in wild compared with captive populations (Sepp et al., 2010). Nevertheless, the ability to detect a negative survival effect of extreme‐low hematocrit in wild populations may be limited by an under‐representation of anemic individuals. Furthermore, hematocrit has been criticized as indicator of ongoing/recent anemia due to disproportionate effect of reticulocytes (Fair et al., 2007; O’Brien et al., 2001). These immature erythrocytes are larger and contain less hemoglobin, meaning hematocrit can recover more rapidly than oxygen‐carrying capacity following anemic episodes. Therefore, anemia could impact survival in wild populations without a detectable change in hematocrit values.

Higher hematocrit was associated with reduced survival probabilities in young individuals, despite nearly all hematocrit values falling within what is considered to be a healthy reference range for captive avifauna, 35%–55% (Campbell, 1994). Short‐term increases in hematocrit can result from dehydration/hemoconcentration, which in turn limit oxygen‐carrying capacity, or increase the cardiovascular effort required to maintain optimal oxygen‐carrying capacity, due to negative relationship between blood viscosity and flow rate. Several studies have observed a lowering of hematocrit (by *ca*. 2%–5%) in birds during endurance activities via an increase in blood plasma (hemodilution). In line with optimal hematocrit theory (Birchard, 1997), these authors suggest that hemodilution is an adaptive response to prolonged exercise, facilitating faster blood flow for less cardiovascular effort (Bury et al., 2019; Jenni et al., 2006; Yap et al., 2018). Thus, high hematocrit in young Seychelles warblers may reflect a failure to maintain optimal hematocrit, for example, due to dehydration. Alternatively, hematocrit could reflect physiological traits and/or life histories with potential costs to survival. For example, hematocrit has been positively associated with reproductive effort (e.g., Hõrak et al., 1998), male ornamentation (Saino et al., 1997), metabolic rate (Yap et al., 2019) and stress (Johnstone et al., 2012). However, further—ideally experiment—studies are needed to confirm the link between hematocrit and pace of life in the Seychelles warbler.

Our study provides novel insights into the dynamics of hematocrit, and its impact on survival, in a wild population. Hematocrit was highly variable within individuals and varied in relation to time of day and (in dominant breeders) breeding stage. This variation limits the utility of hematocrit as a marker of age or senescence. However, the overarching relationship observed with advancing age supports the concept of changing oxygen demands with age. Interestingly, we show that hematocrit can be an indicator of survival prospects in wild populations. Whether survival is directly impacted by (suboptimal) oxygen‐carrying capacity, or factors that increase hematocrit (dehydration, stress, etc.) remains to be tested. Since changes in erythrocyte mass occur over longer timescales than, for example, stress hormones and oxidative stress (Bonier et al., 2009; van de Crommenacker et al., 2017), hematocrit may be a better indicator an individual's baseline stress levels (Johnstone et al., 2012). However, short‐term changes in blood plasma volume can affect hematocrit levels independently of erythrocyte mass, which makes unraveling the drivers of elevated hematocrit difficult without data on additional blood metrics, such as plasma protein and hemoglobin concentrations (see Johnstone et al., 2017). Nevertheless, hematocrit can aid in quantifying physiological state or condition in wild vertebrates, which is a fundamental concept in the study of life‐history trade‐offs.

## CONFLICT OF INTERESTS

None declared.

## AUTHOR CONTRIBUTION

**Thomas James Brown:** Conceptualization (equal); Data curation (lead); Formal analysis (lead); Funding acquisition (equal); Investigation (equal); Methodology (equal); Writing‐original draft (lead); Writing‐review & editing (lead). **Martijn Hammers:** Conceptualization (equal); Funding acquisition (equal); Investigation (equal); Methodology (equal); Writing‐review & editing (equal). **Martin Taylor:** Investigation (equal); Methodology (equal); Supervision (supporting); Writing‐review & editing (equal). **Hannah Dugdale:** Funding acquisition (equal); Investigation (supporting); Methodology (supporting); Supervision (supporting); Validation (equal); Writing‐review & editing (equal). **Jan Komdeur:** Funding acquisition (equal); Writing‐review & editing (equal). **David Richardson:** Conceptualization (equal); Formal analysis (equal); Funding acquisition (equal); Investigation (equal); Methodology (equal); Supervision (lead); Writing‐review & editing (equal).

## ETHICS APPROVAL

All fieldwork was conducted in accordance with local ethical regulations and agreements. The Seychelles Bureau of Standards and Department of Environment gave permission for sampling and fieldwork. Nature Seychelles gave permission to carry out research on Cousin Island.

## Supporting information

Supplementary Material

## Data Availability

Data are available from the Dryad Digital Repository: https://doi.org/10.5061/dryad.7wm37pvrp
