# Supplementary material for: Hematocrit, age, and survival in a wild vertebrate population
Source: Ecol Evol. 2020 Dec 21;11(1):214–26. doi: 10.1002/ece3.7015 (PMC7790625; doi:10.1002/ece3.7015)
Supplement: Supplementary file 1 — Supplementary Material [file ECE3-11-214-s001.pdf]

Supplementary materials

Tables

Table S1: Haematocrit in relation to cross-sectional age and other factors in Seychelles warblers. Results are from a GAMM analysis with a non-parametric smoothing parameter for age. Significant effects are in bold. The analysis includes only individuals with a known age-at-death; shorter-lived individuals had higher haematocrit.

Predictor	β	SE	*t*	*P*
**(Intercept)**	0.464	0.006	74.465	< 0.001
**Sex (Male)**	-0.003	0.003	-0.964	0.335
**Status (Dominant)**	**-0.019**	**0.004**	**-4.786**	**< 0.001**
**Sample Time**	**-0.001**	**0.000**	**-3.646**	**< 0.001**
**Age at death**	**-0.001**	**0.001**	**-2.605**	**0.009**
**Sex × Status**	**0.023**	**0.004**	**5.231**	**< 0.001**
**Smoothed Terms**		***df***	**F**	***P***
**Age**		**7.381**	**13.21**	**< 0.001**
**Random factors**	**854 observations**	**Variance**		
**Individual identity**	466 individuals	< 0.000		
**Breed group**	532 breed groups	< 0.000		
**Catch year**	12 years	< 0.000		

Table S2: Haematocrit in relation to cross-sectional age and other factors in Seychelles warblers. Results are from a GAMM analysis with a non-parametric smoothing parameter for age. Significant effects are in bold. The analysis includes only Sexually mature individuals (≥8 months old) caught outside of known breeding attempts.

Predictor	β	SE	*t*	*P*
**(Intercept)**	0.466	0.008	56.797	< 0.001
**Sex (Male)**	0.001	0.007	0.206	0.837
**Status (Dominant)**	**-0.016**	**0.005**	**-2.990**	**0.003**
**Sample Time**	**-0.002**	**0.001**	**-3.865**	**< 0.001**
**Sex × Status**	**0.020**	**0.008**	**2.511**	**0.012**
**Smoothed Terms**		***df***	**F**	***P***
**Age**		**1.885**	**5.874**	**0.017**
**Random factors**	**374 observations**	**Variance**		
**Individual identity**	297 individuals	< 0.000		
**Breed group**	272 breed groups	< 0.000		
**Catch year**	13 years	< 0.000		

Table S3: Haematocrit in relation to cross-sectional age and other factors in Seychelles warblers. Results are from a GAMM analysis with a non-parametric smoothing parameter for age and time to lay date (days). Significant effects are in bold. The analysis includes only dominant breeder individuals caught within 50 days of known breeding attempts.

**Predictor**	**β**	**SE**	***t***	***P***
**(Intercept)**	0.437	0.007	65.409	< 0.001
**Sex (Male)**	**0.028**	**0.003**	**10.065**	**< 0.001**
**Sample Time**	**-0.001**	**0.000**	**-2.694**	**0.007**
**Smoothed Terms**		***df***	**F**	***P***
**Age**		**1.674**	**3.102**	**0.035**
**Time to lay date (Female)**		**4.05**	**3.211**	**0.014**
**Time to lay date (Male)**		**5.144**	**5.313**	**< 0.001**
**Random factors**	**436 observations**	**Variance**		
**Individual identity**	281 individuals	< 0.000		
**Breed group**	322 breed groups	< 0.000		
**Catch year**	12 years	< 0.000		

Table S4: Haematocrit in relation to cross-sectional age and other factors in Seychelles warblers. Results are from a GAMM analysis with a non-parametric smoothing parameter for age and time to lay date (days). Significant effects are in bold. The analysis includes only sexually mature (≥ 8 months old) subordinate individuals caught within 50 days of known breeding attempts (of the subordinates breed group).

**Predictor**	**β**	**SE**	***t***	***P***
**(Intercept)**	0.468	0.012	40.568	< 0.001
**Sex (Male)**	0.007	0.005	1.223	0.223
**Sample Time**	**-0.002**	**0.001**	**-2.527**	**0.012**
**Smoothed Terms**		***df***	**F**	***P***
**Age**		1.823	2.174	0.209
**Time to lay date (Female)**		**2.253**	**3.445**	**0.032**
**Time to lay date (Male)**		1	0.986	0.322
**Random factors**	**182 observations**	**Variance**		
**Individual identity**	140 individuals	< 0.000		
**Breed group**	140 breed groups	< 0.000		
**Catch year**	12 years	< 0.000		

Table S5: Haematocrit in relation to cross-sectional age (Mean age) and longitudinal age (Δ Age) in Seychelles warbler <1.5 years old and 1.5–13 years old. Parameters shown are from LMM analysis. Significant effects are in bold. Model excludes dominant breeding individuals caught during key breeding stages, where haematocrit deviates from typical levels; 20 days before to 5 days after laying for dominant males, and 30 – 50 days after laying in dominant females.

**< 1.5 YEARS OLD**				
**Predictor**	**β**	**SE**	***t***	***P***
**(Intercept)**	0.392	0.010	40.682	< 0.001
**Mean Age**	**0.160**	**0.022**	**7.351**	**< 0.001**
**Mean Age2**	**0.037**	**0.008**	**4.533**	**< 0.001**
**Δ Age**	**-0.086**	**0.016**	**-5.457**	**< 0.001**
**Δ Age2**	**-0.052**	**0.018**	**-2.968**	**0.003**
**Sex (Male)**	-0.005	0.003	-1.579	0.115
**Status (Dominant)**	**-0.014**	**0.007**	**-2.08**	**0.038**
**Sample Time**	**-0.002**	**0.000**	**-3.272**	**0.001**
**Sex × Status**	0.015	0.008	1.75	0.081
**Random factors**	**625 observations**	**Variance**		
**Individual identity**	499 individuals	< 0.000		
**Breed group**	448 breed groups	< 0.000		
**Catch year**	14 years	< 0.000		
**1.5 - 13 YEARS OLD**				
**Predictor**	**β**	**SE**	***t***	***P***
**(Intercept)**	0.463	0.007	70.789	< 0.001
**Mean Age**	**-0.002**	**0.001**	**-2.899**	**0.004**
**Δ Age**	**-0.002**	**0.001**	**-2.728**	**0.007**
**Sex (Male)**	**0.021**	**0.003**	**7.783**	**< 0.001**
**Status (Dominant)**	**-0.013**	**0.004**	**-3.539**	**< 0.001**
**Sample Time**	**-0.002**	**0.000**	**-3.681**	**< 0.001**
**Random factors**	**625 observations**	**Variance**		
**Individual identity**	372 individuals	< 0.000		
**Breed group**	469 breed groups	< 0.000		
**Catch year**	14 years	< 0.000		

Table 3. Survival in the Seychelles warbler in relation to haematocrit for individuals 1.5 years old and 1.5–13 years old. Results are from binominal GLMMs with survival to the following year (Y/N) as the response variable. Significant effects are in bold. Model excludes dominant breeding individuals caught during key breeding stages, where haematocrit deviates from typical levels; 20 days before to 5 days after laying for dominant males, and 30 – 50 days after laying in dominant females.

**< 1.5 YEARS OLD**				
**Predictor**	**β**	**SE**	***z***	***P***
**(Intercept)**	5.114	1.284	3.981	< 0.001
**Haematocrit**	**-9.565**	**3.009**	**-3.179**	**0.001**
**Sex (Male)**	-0.236	0.223	-1.057	0.291
**Status (Dominant)**	0.394	0.376	1.048	0.295
**Age**	0.502	0.397	1.264	0.206
**Random factors**	**497 observations**	**Variance**		
**Breed group**	414 breed groups	< 0.000		
**Catch year**	14 years	0.327		
**1.5 - 13 YEARS OLD**				
**Predictor**	**β**	**SE**	***z***	***P***
**(Intercept)**	1.688	1.816	0.929	0.353
**Haematocrit**	-1.692	4.012	-0.422	0.673
**Sex (Male)**	-0.489	0.267	-1.833	0.067
**Status (Dominant)**	**0.707**	**0.349**	**2.024**	**0.043**
**Age**	-0.028	0.045	-0.634	0.526
**Random factors**	**348 observations**	**Variance**		
**Catch year**	13 years	0.238		

Figures

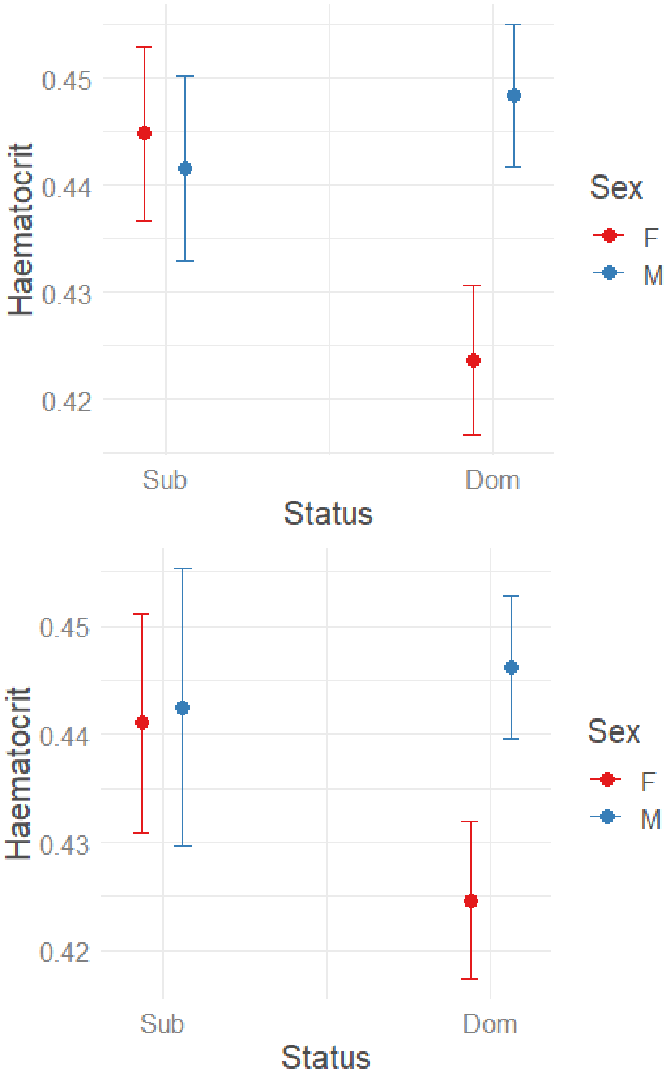

Figure S1) Haematocrit in relation to sex and status of Seychelles warblers. Points are model predicted haematocrit ± 95% CI, adjusted for age and time of sampling (held at sample average). The top plot includes all individuals of all ages, irrespective of breeding stage (*N* = 1379). The bottom plot only includes sexually mature (≥8 months old) individuals caught outside of known breeding attempts (*N* = 374). Sub = Subordinate, Dom = Dominant breeder.

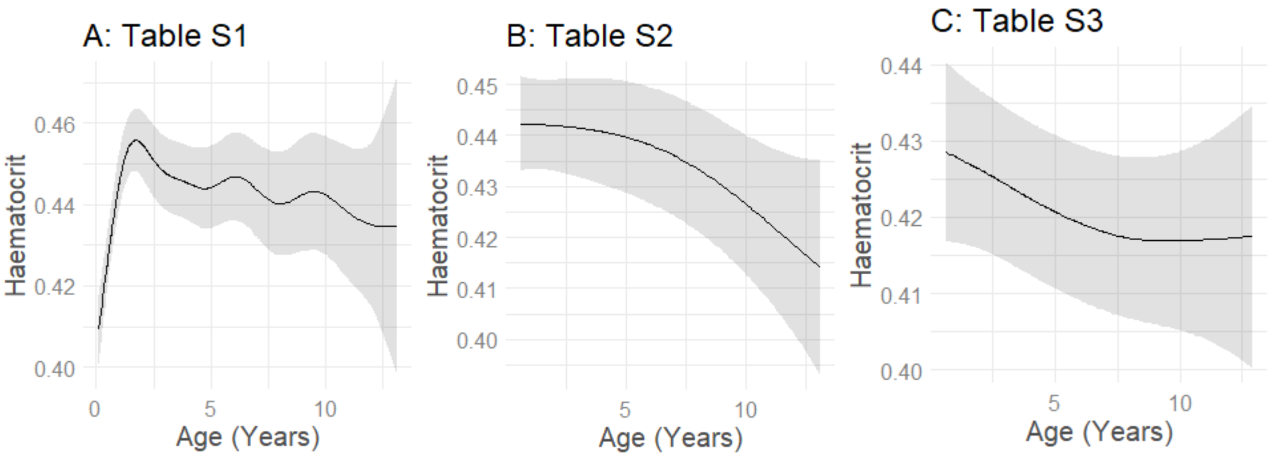

Figure S2 – Model predicted haematocrit in relation to age in Seychelles warblers. Plots are derived from models summarised in tables S1, S2 and S3. Datasets include; A) All individuals that died before the time of analysis (controlling for age-at-death); B) All sexually-mature (> 8 months-old) sampled outside of known breeding attempts; c) All dominant individuals sampled during breeding attempts (controlling for days from lay date).
